# Automating thematic review of prevention of future deaths reports: concordance study of a child-suicide analysis using large language models

**DOI:** 10.1136/bmjment-2025-302212

**Published:** 2026-02-25

**Authors:** Sam Osian, Arpan Dutta, Sahil Bhandari, Iain E Buchan, Dan W Joyce

**Affiliations:** 1 Department of Primary Care and Mental Health, Civic Health Innovation Labs and Mental health Research for Innovation Centre, University of Liverpool, Liverpool, UK; 2 Mental health Research for Innovation Centre, Mersey Care NHS Foundation Trust, University of Liverpool, Liverpool, UK; 3 Civic Health Innovation Labs and Mental health Research for Innovation Centre, University of Liverpool, Liverpool, UK

**Keywords:** Adolescent, Mental Health, Mental Health Services

## Abstract

**Background:**

Prevention of future deaths (PFD) reports issued by coroners in England and Wales identify systemic safety hazards but are difficult to analyse at scale. Reports are not machine-readable, lack consistent metadata and cannot be reliably searched or exported, meaning prior national reviews have relied on labour-intensive manual screening and coding.

**Objective:**

To evaluate whether a fully automated, vision-enabled large language model (LLM) pipeline (PFD Toolkit) can replicate and extend the Office for National Statistics (ONS) thematic review of child-suicide PFD reports, and to assess concordance with blinded clinical adjudication.

**Methods:**

All PFD reports published between July 2013 and November 2023 (n=4730) were scraped from judiciary.uk and processed using PFD Toolkit, which combines optical character recognition with LLM-powered screening and thematic coding. Reports were classified for child suicide (≤18 years), addressee categories and 23 coroner-concern subthemes mirroring the ONS coding frame. Agreement was evaluated against a blinded clinical reference standard: three psychiatrists independently adjudicated a stratified sample of 146 reports (73 Toolkit-positive cases and 73 decoys), with disagreements resolved by consensus. Inter-rater reliability and index-reference agreement were quantified using kappa statistics.

**Findings:**

The Toolkit identified 73 child-suicide PFD reports between July 2013 and November 2023, compared with 37 identified in the ONS review. 62 cases fell within the ONS analytical window, and 11 pre-dated the introduction of suicide-related tags on the PFD archive. Pre-consensus inter-rater reliability among clinicians was substantial to almost perfect (Fleiss’ κ=0.75, 95% CI 0.65 to 0.84). Post-consensus agreement between the Toolkit and the clinical reference standard was substantial to almost perfect (Cohen’s κ=0.93, 95% CI 0.77 to 1.00; raw agreement 97%). End-to-end screening, coding and tabulation of all reports completed in 5 min 29 s on a consumer-grade laptop.

**Conclusions:**

A national thematic review of child-suicide PFD reports can be fully automated with high concordance to expert judgement, dramatically reducing time and labour while recovering previously missed cases.

**Clinical implications:**

Automated analysis of PFD reports enables rapid, reproducible surveillance of recurring system failures, supporting more timely public health intelligence, policy responses and learning from coronial data.

**WHAT IS ALREADY KNOWN ON THIS TOPIC**

- Prevention of future deaths (PFD) reports capture coroners’ safety concerns but are difficult to analyse at scale because the archive lacks consistent, structured metadata and offers no means to create or export custom annotated datasets, with many reports only available as scanned documents. Prior work has therefore relied on manual, labour-intensive screening and coding.

**WHAT THIS STUDY ADDS**

- An automated pipeline (PFD Toolkit) replicated the Office for National Statistics (ONS) thematic analysis of child-suicide cases, finding 73 relevant reports (vs 37 in the ONS analysis), showing substantial to almost-perfect agreement with blinded clinical adjudication, in around 5 min of runtime (compared with what would otherwise take months of manual review).

**HOW THIS STUDY MIGHT AFFECT RESEARCH, PRACTICE OR POLICY**

- This approach enables rapid, automated and reproducible surveillance to identify recurring safety concerns contained within coroners’ PFD reports.

## Introduction

 Prevention of future deaths (PFD) reports are statutory notices from coroners in England and Wales, highlighting circumstances that may risk further deaths. Each PFD report describes the context of an inquest, details factors believed to have contributed and lists concerns that the coroner believes the recipients of the reports are well placed to address.

These documents represent a unique window into ground-level public-safety hazards. However, despite being publicly available on the judiciary.uk website,^1^ the research potential of PFD reports has remained largely under-exploited, primarily due to practical barriers to access and analysis. Reports are dispersed across a poorly indexed judicial website, lack consistent metadata and are often available only as scanned images without searchable (machine-readable) text. No downloadable dataset is available for systematic retrieval. Although the online repository provides category-based filters (such as care home deaths or deaths in hospitals), these are inconsistently applied; around 70% of reports lack any category-based tag altogether.^2^

Consequently, even high-profile research has relied on labour-intensive manual screening and annotation, demanding months or even years of researcher capacity.^3 4^ The House of Commons Justice Committee conceded in 2021 that this system is ‘under-developed’ for public safety, citing the lack of ability to search and analyse reports to identify recurring issues.^5^

The Office for National Statistics (ONS) has offered the first systematic study of PFD reports related to child suicide.^6^ The study involved the manual screening of hundreds of reports, identifying 37 child-suicide cases published between January 2015 and November 2023. The ONS researchers also manually screened, reviewed and coded reports by recipient, as well as 23 ‘coroner concerns’ topics across six broad themes, from service-provision failures to gaps in communication and staffing. Candidate reports were identified via the Courts and Tribunals Judiciary website categorisations (‘Child death (from 2015)’ plus ‘Suicide (from 2015)’ and/or ‘Mental health related death’), then manually downloaded, screened and thematically coded. The authors noted that reliance on these categorisations may miss relevant reports as categorisation on the judiciary.uk archive is incomplete and inconsistently applied.

To address the manual burden associated with studies such as these, there is a need for tools that can reliably and efficiently process the entire PFD corpus, including scanned or untagged reports, without relying on inconsistent manual filters or metadata. An ideal solution would automate tasks that currently require manual effort, including screening for relevant cases, discovering themes and generating user-defined metadata (eg, age of the deceased) for each report.

Recent advances in large language models (LLMs), including multimodal models capable of image ingestion, offer a way to overcome these constraints. We developed the PFD Toolkit, an open-source Python package for automated extraction of structured data from unstructured PFD reports (ie, ‘text-to-table’).^7^ The PFD Toolkit integrates LLMs and optical character recognition (OCR) web scraping, along with LLM-driven topic discovery, to enable systematic interrogation of the entire PFD corpus, regardless of file format or quality. This automated approach enables highly scalable and reproducible research pipelines, in contrast to the lengthy and opaque processes previously required.

By applying the PFD Toolkit’s automated vision-LLM pipeline to the entire corpus of 4730 PFD reports (July 2013 to November 2023), we replicate the ONS coding schema at scale, recover missed cases and benchmark performance against post-consensus clinical annotations. This offers a comprehensive and reproducible alternative to months-long manual review.

This work complements initiatives such as the Preventable Deaths Tracker (PDT),^8^ which enhances access and searchability of PFD reports (https://preventabledeathstracker.net/). Unlike the PDT, which deals only in metadata, PFD Toolkit processes both metadata and the long-text content of each report. It also provides an end-to-end, automated workflow for user-defined screening and thematic coding, supporting scalable, efficient and reproducible research applications.

To improve accessibility for non-programmers, we also developed PFD Toolkit Workbench (https://workbench.pfdtoolkit.org), a browser-based interactive environment that provides the same automated screening and coding functionality through a graphical interface.

## Methods

### Study design

**Figure 1:**
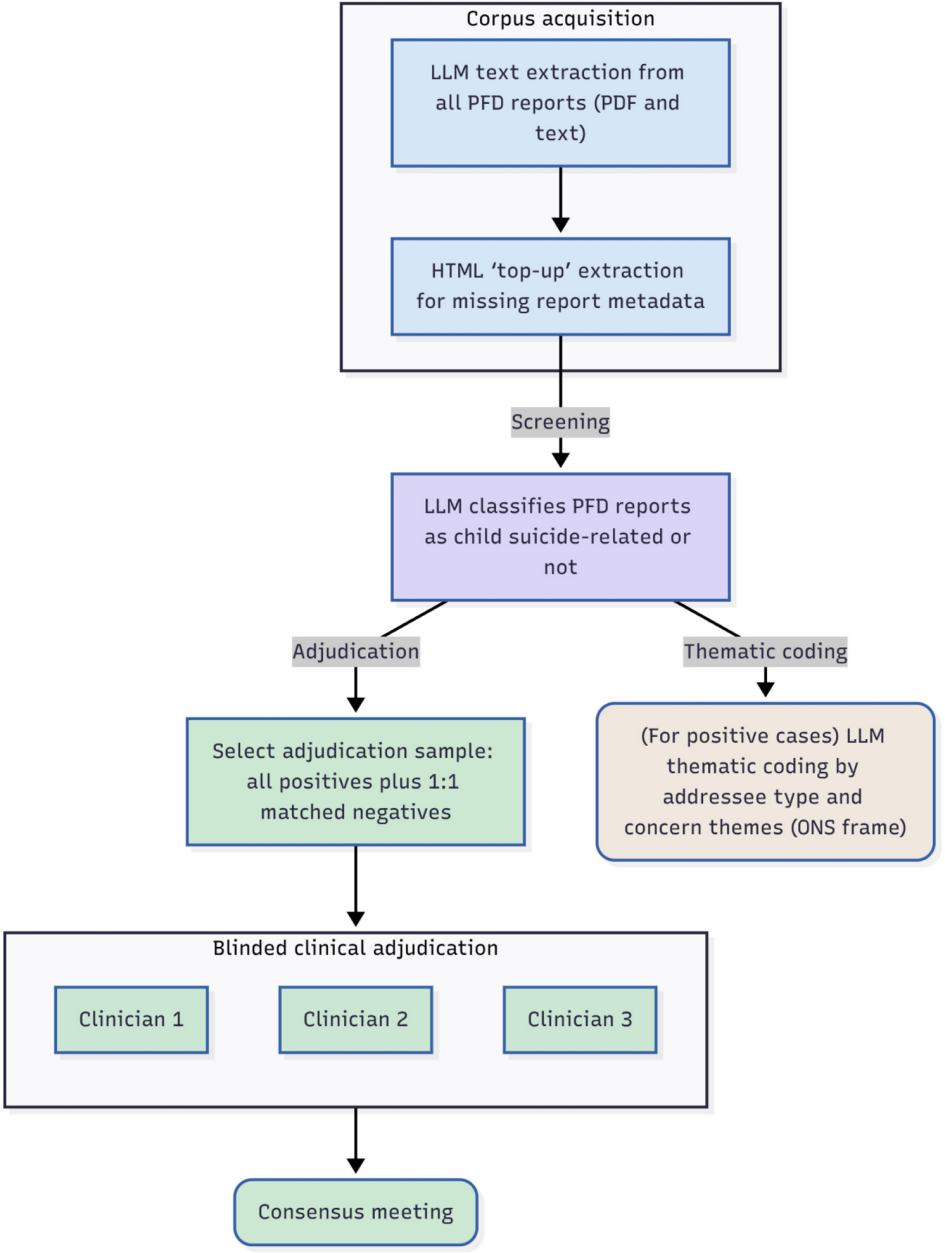
Workflow diagram for corpus acquisition, screening, thematic coding and clinical adjudication stages of the study. LLM, large language models; ONS, Office for National Statistics; PFD, prevention of future deaths.

We conducted an agreement (reliability/concordance) study comparing PFD Toolkit’s automated LLM pipeline with a blinded clinical reference standard on PFD reports (with individual reports as the unit of analysis). Reporting follows the Guidelines for Reporting Reliability and Agreement Studies.^9^ The pipeline, implemented in PFD Toolkit V.0.3.7 (figure 1), handled ingestion, screening, coding and tabulation. Outputs were benchmarked against two reference standards: (1) the total number of reports identified in the published ONS study (n=37) and (2) an independent, blinded clinical review of a stratified, decoy-boosted sample of 146 reports, used as a gold standard to measure agreement.

The primary outcome was kappa agreement for identified reports against a clinical reference standard. Secondary outcomes include the number of child-suicide PFD reports identified, compared with the ONS’s manual review and end-to-end script processing wall-clock time.

### Corpus acquisition

The PFD Toolkit scraped all reports under the ‘prevention-of-future-death-reports’/path from the judiciary.uk website. The Toolkit processed both text-based and scanned documents using a multistage pipeline that includes 200 dpi Base64 image conversion and vision-enabled LLM text extraction via OCR, followed by HTML scraping as a fail-safe to retrieve any missing report-level metadata. Further details of the design and implementation of the PFD Toolkit are described elsewhere (https://github.com/Sam-Osian/PFD-toolkit).

From the judiciary.uk archive, we assembled a base corpus of 4730 unique PFD reports published July 2013 to November 2023. All index-positive and index-negative (‘decoy’) items for adjudication were sampled from this same window.

The ONS review, by contrast, drew its sample from reports categorised on the judiciary.uk website as ‘Child death (from 2015)’ with an additional categorisation of ‘Suicide (from 2015)’ and/or ‘Mental health related death’. This constrained the analytical window to January 2015 to November 2023 and may have missed relevant cases where categories were absent or inconsistently applied. Because the PFD Toolkit does not depend on judiciary.uk categorisation and instead extracts and codes report content directly, it can identify relevant cases published before 2015; we therefore included reports published prior to January 2015 in our analysis.

### Units of analysis and selection

The unit of measurement was report-level classification and coding (child-suicide yes/no; addressee categories; concern subthemes). For agreement testing, the positive stratum comprised a census of all reports that the PFD Toolkit classified as child suicide cases within the analytical window (July 2013 to November 2023; n=73). The negative stratum comprised a simple random sample drawn without replacement from the entire remaining archive of reports the index method classified as not child suicide cases (n=73), within the same analytical window. The 1:1 balance between index-positive and ‘decoy’ cases was prespecified by SO to limit expectation bias of raters, and as such was not communicated to raters until fieldwork completion.

Each blind, independent clinical rater was presented with 146 PFD reports and was required to decide whether each PFD report was either (1) describing a child (aged ≤18 years at the date of death) and that they had died by suicide or (2) a decoy case where the PFD report referred to someone who was either not a child or did not die by suicide. Raters were blinded to index outputs and each other’s initial assessments until the consensus stage.

### Screening and thematic coding

We prompted GPT-4.1^10^ via PFD Toolkit to code reports by: (1) relevance to child suicide (inclusive of those aged 18, allowing inference from cues such as Child and Adolescent Mental Health Services (CAMHS) involvement or school year where age was not explicitly recorded), (2) addressee categories as per the ONS coding frame: National Health Service body; government department or minister; local authority; professional body; other. (The ONS did not define ‘professional body,’ and so our research adopted the following definition: ‘an organisation with statutory responsibility for a profession, such as the GMC, NMC, Royal Colleges’.) (3) Coroner concerns: 23 subthemes under six headline themes as per the ONS coding frame: service provision; staffing and resources; communication; multiple services involved in care; accessing services; access to harmful content and environment.

For each stage, outputs were coerced into JSON arrays containing Boolean values, enforced and validated with Pydantic.^11^ To support transparency, the Toolkit can optionally output supporting text evidence for each coded item. Although automated, the approach therefore remains open to human-in-the-loop workflows or post hoc review as needed.

### Rater population and procedures

The number of raters was determined using fixed-n binary kappa power analysis. With an anticipated kappa of 0.70, the lower bound of the 95% CI was estimated as 0.589 for two raters, 0.617 for three, 0.626 for four and 0.630 for five. Since three raters exceeded the conventional ‘substantial agreement’ threshold (≥0.61),^12^ and additional raters offered only minor improvements, a panel of three was deemed sufficient for this concordance study.

The desired reference standard population included trained psychiatrists with (1) full membership of the Royal College of Psychiatrists, and (2) experience of serious-incident reviews and the UK coronial inquest system. Three psychiatrists (AD, SB and DWJ) were recruited as a convenience sample reflecting that population.

Each rater was tasked with independently rating each sampled report as a child-suicide case or not, blinded to the index outputs and to each other’s assessments. Raters received brief guidance on the aims and objectives of the study prior to review. The index method processed the full corpus before sampling. Raters then assessed the sampled items in randomised order. Raters evaluated the same textual content that informed the index classifications (full report text). As the materials are archival documents, there was no temporal change between index and reference assessments.

For ambiguous cases, the raters were instructed to use the ‘balance of probabilities’ standard for judging if the report related to a child suicide; for example, if suicidal intent was not explicitly recorded in the PFD report, clinicians were asked to review the PFD report text and decide. Disagreements between the three independent raters were resolved at a consensus meeting to form the final reference labels.

### Statistical methods

The primary agreement measures were Cohen’s kappa (index vs consensus reference) and overall percentage agreement. Cohen’s kappa was computed as unweighted (nominal) using the function *kappa2* from the *irr* R package.^13^ Fleiss’ kappa for the three independent raters before consensus was computed using the function *kappam.fleiss* from the *irr* package. For both kappa estimates, two-sided 95% Wald CIs were derived from the test statistics returned by the *irr* package, recovering the SE as kappa divided by z and forming kappa ±1.96×SE. Raw agreement was calculated as the proportion of identical classifications. No report ingestion or parse failures occurred, and all raters reached consensus on every item; therefore, procedures for handling indeterminate data were not required.

### Computational environment

Analyses were run on a consumer-grade (1 x i9-13900H CPU and 64 GB of RAM) Ubuntu V.24.04 laptop. LLM calls to GPT-4.1 were handled by OpenAI’s cloud Application Programming Interface (therefore, we did not require a Graphics Processing Unit, GPU, to execute PFD Toolkit). PFD Toolkit also supports local LLM frameworks (eg, Ollama) as an alternative to proprietary, server-side tooling.

## Results

### Report identification

The PFD Toolkit identified 73 child-suicide PFD reports published between July 2013 and November 2023, compared with 37 identified in the ONS study covering January 2015 to November 2023 (table 1). Of the Toolkit-identified cases, 62 fell within the ONS time frame, while 11 were published prior to the introduction of ‘suicide’ and ‘child death’ categories on the judiciary.uk website in January 2015.

**Table 1:** Count of child-suicide PFD reports identified by PFD Toolkit and ONS

Period	PFD Toolkit	ONS
January 2015 to November 2023 (ONS window)	62	37
Pre-2015	11	–
Total	73	37

ONS, Office for National Statistics; PFD, prevention of future deaths.

For agreement testing, a total of 146 items were assessed: 73 index-positive (ie, child suicide) cases from July 2013 to November 2023, and 73 decoy (ie, not child suicide) cases drawn at random from the same time period. The median publication year of all reports was 2018 (IQR 2016–2021), with similar distributions for index-positive cases (median 2018, IQR 2016–2021) and decoy cases (median 2019, IQR 2016–2022).

### Clinical adjudication

All 146 reports received independent ratings from each of the three raters. 27 instances of disagreement between initial ratings were escalated to consensus where a final label was provided. There were no instances of raters unable to reach a consensus for any given report. No index parse failures/indeterminates occurred.

Prior to consensus, inter-rater reliability among the three clinical raters was Fleiss’ κ=0.745 (95% CI 0.652 to 0.839), indicating substantial to almost-perfect agreement. (According to the widely cited Landis and Koch scale, kappa values between 0.61 and 0.80 can be interpreted as ‘substantial agreement’, while values greater than 0.80 indicate ‘almost perfect agreement’*.*^12^)

Post-consensus, agreement between the PFD Toolkit (index) and the clinical reference standard was Cohen’s κ=0.93 (95% CI 0.77 to 1.00); raw agreement 97%.

### Run time

End-to-end processing wall-clock time was 5 min 29 s. This included screening the entire corpus (n=4730) for child-suicide relevance, thematic coding and tabulation.

### Thematic outputs

Tables2  3 mirror the ONS thematic outputs on addressees and coroner concern subthemes. In keeping with the ONS reporting structure, we present addressee distributions (table 2) separately from coroner concern subthemes (table 3), as these describe different dimensions: recipient entities versus issue types raised. Because a single report may have multiple addressees or subthemes, totals exceed the number of reports analysed. Note that the ONS counted ‘mentions’ within and across reports, whereas PFD Toolkit reports presence at the report level, so counts cannot be directly compared with the corresponding figures in the ONS bulletin.

**Table 2:** Addressee categories coded by PFD Toolkit (n=73 reports)

Category	Count of reports
NHS trust or CCG	40
Government department or minister	31
Local authority	15
Professional body	8
Other	28

CCG, Clinical Commissioning Group; PFD, prevention of future deaths.

**Table 3:** Coroner-concern subthemes coded by PFD Toolkit (n=73 reports)

Theme/subtheme	Count of reports
Service provision	
Standard operating procedures or processes not followed or adequate	34
Risk assessment	23
Specialist services (crisis, autism, beds)	18
Discharge from services	4
Diagnostics	8
Staffing and resources	
Training missing, inadequate or not mandatory	25
Inadequate staffing	12
Lack of funding	8
Recruitment and retention problems	4
Communication	
Lack of communication between services	25
Within-service communication is poor	24
Lack of communication with patient and family	18
Confidentiality risk not communicated	9
Multiple services involved in care	
Issues with local authority (including children’s services, schools)	15
Integration of care was disconnected	20
Transition from CAMHS to adult services ineffective	14
Accessing services	
Delays in referrals and waiting times	24
Patient engagement lacking	8
Referrals rejected	5
Access to harmful content and environment	
Access to harmful items or substances	9
Internet content and controls	4
Access to trainlines	3
Safeguarding from sensitive material	4

CAMHS, Child and Adolescent Mental Health Services; PFD, prevention of future deaths.

## Discussion

### Principal findings

This study demonstrates that a national thematic review of coroners’ reports can be fully automated without compromising reliability or validity. Using a vision-enabled, LLM-based workflow, the PFD Toolkit reproduced expert-derived outputs with substantial to almost-perfect agreement. It completed analysis in minutes, compared with resource-intensive screening, curation and analyses requiring months of human resource. Compared with the ONS study, the Toolkit identified nearly two times as many child-suicide PFD reports, applying a consistent coding frame across all cases. By turning unstructured coronial text into structured, machine-readable data, this approach converts a static archive into a living, continuously analysable dataset—establishing the technical basis for routine, reproducible and actionable surveillance of preventable deaths.

### Limitations

Coroners are not required to record the age of the deceased in PFD reports, and in practice, age may only be included when considered contextually relevant. While we believe that child status is likely to be documented in most suicide cases, it remains possible that some relevant cases were missed if the coroner did not specify the deceased’s age. However, this is a data quality/completeness issue, rather than a limitation of PFD Toolkit itself.

The performance of the PFD Toolkit is also likely to depend on the specific language model used for extraction and coding. This research used OpenAI’s GPT-4.1 via the cloud API. Different LLMs may yield varying levels of accuracy and efficiency, and future updates or alternative models may produce different results.

Because the ONS did not release their annotated report-level dataset, we were unable to directly compare our screening results on a report-by-report basis. Additionally, the ONS counted each mention of a concern within and across reports, whereas our approach reports the presence of each concern at the report level. This difference in counting methods limits the comparability of subtheme frequencies between the two analyses.

During peer review, the original ONS digital bulletin was published as a peer-reviewed article in the *British Journal of Psychiatry*.^6^ Compared with the original bulletin, the subsequent peer-reviewed article includes additional descriptive tables beyond the thematic outputs reproduced here.

### Implications

Beyond the present case study, the PFD Toolkit provides a generalisable infrastructure for research and policy work using PFD reports. By automating ingestion, screening and thematic coding across the full archive (including scanned reports), it lowers manual burden, enables rapid reviews and routine surveillance across different coronial areas and supports analyses of longitudinal trends. The same pipeline can be configured for diverse use cases (eg, deaths in custody, medication safety, care-home incidents, maternal mortality), making large-scale analyses feasible within an otherwise under-developed system.

A persistent barrier to national learning is that PFD reports lack a shared ontology and a consistent, machine-readable schema. Important entities (eg, age, sex, care setting, services and agencies involved, concern types) are expressed idiosyncratically across reports, limiting reliable recall, comparability and reproducibility. The PFD Toolkit addresses this gap by enabling users to define and apply their own structured ontology at scale: configurable extraction and coding templates automatically label the corpus with new columns corresponding to user-defined variables, transforming unstructured coronial narratives into consistent tabular data suitable for quantitative analyses. This positions the Toolkit not only as an automation approach but as a practical scaffold for standardisation across future PFD analyses. PFD Toolkit includes additional capabilities which may prove useful for research and policy work. It can output snippet-level text evidence for each coded item, supporting transparent trace-back and internal quality assurance; generate concise report summaries to accelerate case review; and offer data-driven theme discovery via a topic-modelling pipeline that can complement or precede prespecified coding frames. These software features were not the focus of the present evaluation, but they strengthen the Toolkit’s utility for routine surveillance and exploratory analyses using coronial data.

## Data Availability

Data are available in a public, open access repository.
